# Ethnic disparities in cardiac transplantation: opportunities to improve long-term outcomes in all cardiac transplant recipients

**DOI:** 10.1186/s13584-019-0320-x

**Published:** 2019-06-11

**Authors:** Jeremy Kobulnik, Yasbanoo Moayedi, Douglas Greig

**Affiliations:** 1 University of Toronto, Sinai Health System and Toronto General Hospital, 600 University Ave, Toronto, Ontario M5G1X5 Canada; 2 Stanford University, Palo Alto, USA; 3 Pontifical Catholic University of Chile, Santiago, Chile

**Keywords:** Cardiac transplantation, Cardiac allograft vasculopathy, Cardiovascular mortality, Ethics, Ethnicity

## Abstract

Ethnic disparities in cardiovascular outcomes have been increasingly recognized in the medical literature. In a recent paper in this journal, Peled et al. provide evidence that Arab Israelis may have worse outcome after cardiac transplant than their Jewish counterparts. This commentary explores possible explanations for the differing outcomes and suggests potential solutions that may improve outcomes for cardiac transplant recipients regardless of ethnicity.

It was with great interest that we read the recent publication entitled “Ethnic disparity in Israel impacts long-term results after heart transplantation”. From a medical perspective, examining disparities of outcomes between subgroups of patients receiving a heart transplant can be a useful exercise to identify modifiable predictors of risk that can then improve future outcomes. From an ethics perspective, it is critical to examine the results of this study for any systematic differences in treatment that may unfairly impact an ethnic minority. As the authors point out, similar differences among ethnic groups have been found in the United States [1].

Peled et al. found that Arab Israelis are at higher risk of cardiac allograft vasculopathy (CAV) and cardiovascular mortality after cardiac transplant compared to Jewish Israelis, despite the same access to a publicly funded universal health care system.

Three important issues need to be closely examined. 1) Is this difference merely a consequence of differing patient populations and relatedly different mechanisms of death? 2) Is the difference related to different medical therapy, including statin use? 3) Does this analysis actually detect discrimination at the level of patient selection rather than during post transplant treatment?

The authors acknowledge that these two groups of patients suffering from advanced heart failure who subsequently received a heart transplant were not otherwise identical. Jewish Israelis were older and more often male. While not statistically significant, Jewish Israelis were numerically more likely to be suffering from heart failure related to coronary artery disease than their Arab counterparts. After adjusting for older age and male gender, CAV rates and cardiovascular mortality were much lower amongst Jewish Israelis.

One possible explanation for this might be competing mortalities. In the discussion, the authors acknowledge that overall mortality did not differ between Jewish Israeli and Arab Israeli recipients. Is it possible that Jewish Israelis were dying from non-graft related issues? Were the Jewish Israelis more likely to be sicker recipients with non-cardiac related co-morbidities such that they did not live long enough to experience CAV or to die from graft dysfunction? Malignancy history, lung function testing and overall frailty for recipients was not provided, though hepatic and renal function were seemingly similar. Jewish Israelis were indeed hospitalized significantly longer prior to transplantation despite similar listing status, sensitization, body size and blood group, potentially hinting at co-morbidities necessitating hospitalization while waiting for transplant.

Jewish Israelis were more likely to be treated with statins than Arab Israelis post transplant and thus had a lower average low density liporotein level 3 months post transplant. It is unclear why this was the case, though it presumably relates to the numerically higher incidence of pre-transplant coronary artery disease and dyslipidemia. Regardless, the authors adjusted for this in their analysis. However, it is not clear that there was any adjustment for the timing of the prescription or the dose prescribed. Statin therapy is a critical piece of the management of cardiac transplantation with pleotropic effects and a landmark study that demonstrated a reduction in hemodynamically significant rejection and improved long-term outcomes in those treated with statin therapy [2, 3]. There is also evidence that the timing of the introduction of statins is important with those being started earlier having lower incidence of CAV [4]. Current guidelines recommend statins for all patients beginning at 1–2 weeks post cardiac transplant, regardless of lipid levels [5].

It is conceivable that Arab Israelis not only were less likely to be treated with statins but also with delayed initiation and at lower doses, possibly related to the misconception that statin use is only beneficial to those with hyperlipidemia and established pre-transplant vascular disease. This may have contributed to the higher incidence of CAV and cardiovascular mortality in Arab Israelis. We would encourage the authors to re-examine their data to see whether they may have evidence that high potency statins are associated with a reduction in CAV and cardiovascular mortality in transplant patients. We would also encourage the authors to look at differences in post transplant aspirin use, as there is emerging data that it can also reduce the risk of developing CAV [6, 7].

We would be remiss if we did not discuss potential ethical implications of this study. It is clear from our previous comments and those of the authors that the differences in outcomes between Arab Israelis and Jewish Israelis may relate to medical and genetic differences, including the ethnic donor/recipient mismatch. However, once dramatic differences in outcomes are identified between different ethnic groups, it is critical to explore any possible correctable systematic discrimination exposing one group to inferior outcomes compared to the other. The authors acknowledge that in general, Israeli Arabs have a lower socioeconomic status than Jewish Israelis, which is a known independent risk factor for worse outcomes in heart failure and cardiac transplant [8, 9]. Ideas for correcting this inequity are beyond the scope of this commentary. And while it is possible that Arab Israelis are in some way systematically treated inferiorly after cardiac transplant (in addition to differing statin/aspirin prescriptions), overall we feel that if present, discrimination is far more likely to occur at the time of referral and listing.

While there are medical guidelines, the decision of whom to list can be subjective, especially as it relates to relative medical contraindications and psychosocial factors. The description of the two patient populations, with the Jewish Israelis being older and potentially sicker with longer pre-transplant hospitalizations, raises concerns that older Arab Israelis with co-morbidities may have been more likely to be declined for cardiac transplant listing. This may have led to transplanted Arab Israelis being more likely to die from graft dysfunction and Jewish Israelis from non-cardiovascular causes. Another equally problematic possibility is that Arab Israelis were overall less likely to be referred for cardiac transplant leading to only young Arab Israelis without co-morbidities being seen in a transplant center. The potential disparity to access to cardiac transplantation is further supported by the fact that Arab Israelis made up only 11% of the transplanted cohort but make up about 20% of the Israeli population; this occurred despite the fact that advanced heart failure should be more prevalent amongst Arab Israelis, given their lower socioeconomic status [9].

## Conclusion

The finding that Arab Israeli’s have a higher incidence of CAV than Jewish Israelis is intriguing and potentially highlights the critical role statins have in cardiac transplant patients. Moreover, it exposes the possibility of a selection bias occurring during cardiac transplant listing decisions. Making sure that all patients suitable for cardiac transplant, regardless of ethnicity and socioeconomic status are actually referred is notoriously difficult. Avoiding bias during patient selection requires being religiously consistent when evaluating relevant information regarding potential transplant candidates. We continue to work on this at our institutions by using specific and consistent selection criteria and a diverse cardiac transplant team.

We commend the authors for examining a sensitive issue in their country and for having the courage to publish their results. We would be delighted to see them continue to study the reasons for differing outcomes amongst Jewish and Arab Israelis and then advocate for measures that would improve outcomes for all Israelis suffering from advanced heart failure.

## Data Availability

Not applicable.

## Abbreviations

CAV: Cardiac allograft vasculopathy
